# Surveillance for respiratory and diarrheal pathogens at the human-pig interface in Sarawak, Malaysia

**DOI:** 10.1371/journal.pone.0201295

**Published:** 2018-07-27

**Authors:** Laura K. Borkenhagen, Kerry A. Mallinson, Rick W. Tsao, Siaw-Jing Ha, Wei-Honn Lim, Teck-Hock Toh, Benjamin D. Anderson, Jane K. Fieldhouse, Sarah E. Philo, Kuek-Sen Chong, William G. Lindsley, Alejandro Ramirez, James F. Lowe, Kristen K. Coleman, Gregory C. Gray

**Affiliations:** 1 Duke Global Health Institute, Duke University, Durham, North Carolina, United States of America; 2 Division of Infectious Disease, School of Medicine, Duke University, Durham, North Carolina, United States of America; 3 SEGi University Sibu Clinical Campus, Sibu, Sarawak, Malaysia; 4 Department of Paediatrics, Sibu Hospital, Sibu, Sarawak, Malaysia; 5 Clinical Research Center, Sibu Hospital, Sibu, Sarawak, Malaysia; 6 Divisional Health Office, Sibu, Sarawak, Malaysia; 7 National Institute for Occupational Safety and Health, Morgantown, West Virginia, United States of America; 8 Department of Veterinary Diagnostics and Production Animal Medicine, College of Veterinary Medicine, Iowa State University, Ames, Iowa, United States of America; 9 Integrated Food Animal Management Systems, Department of Veterinary Clinical Medicine, College of Veterinary Medicine, University of Illinois at Urbana-Champaign, Champaign, Illinois, United States of America; 10 Duke-NUS Medical School, Singapore, Singapore; Nanjing Agricultural University, CHINA

## Abstract

**Background:**

The large livestock operations and dense human population of Southeast Asia are considered a hot-spot for emerging viruses.

**Objectives:**

To determine if the pathogens adenovirus (ADV), coronavirus (CoV), encephalomyocarditis virus (EMCV), enterovirus (EV), influenza A-D (IAV, IBV, ICV, and IDV), porcine circovirus 2 (PCV2), and porcine rotaviruses A and C (RVA and RVC), are aerosolized at the animal-interface, and if humans working in these environments are carrying these viruses in their nasal airways.

**Study:**

This cross-sectional study took place in Sarawak, Malaysia among 11 pig farms, 2 abattoirs, and 3 animal markets in June and July of 2017. Pig feces, pig oral secretions, bioaerosols, and worker nasal wash samples were collected and analyzed via rPCR and rRT-PCR for respiratory and diarrheal viruses.

**Results:**

In all, 55 pig fecal, 49 pig oral or water, 45 bioaerosol, and 78 worker nasal wash samples were collected across 16 sites. PCV2 was detected in 21 pig fecal, 43 pig oral or water, 3 bioaerosol, and 4 worker nasal wash samples. In addition, one or more bioaerosol or pig samples were positive for EV, IAV, and RVC, and one or more worker samples were positive for ADV, CoV, IBV, and IDV.

**Conclusions:**

This study demonstrates that nucleic acids from a number of targeted viruses were present in pig oral secretions and pig fecal samples, and that several viruses were detected in bioaerosol samples or in the nasal passages of humans with occupational exposure to pigs. These results demonstrate the need for future research in strengthening viral surveillance at the human-animal interface, specifically through expanded bioaerosol sampling efforts and a seroepidemiological study of individuals with exposure to pigs in this region for PCV2 infection.

## Introduction

Industrialized livestock operations contain dense populations of animals and humans that are frequently in close contact. As these practices have become more prevalent, the risk of emerging zoonotic diseases has escalated [1–3]. This is evidenced by the 2009 Influenza A (H1N1) pandemic virus which contained genetic components from swine-reservoired viruses, and rapidly spread to at least 214 countries causing at least 151,700 human deaths within a cumulative 12 months of outbreak in each country [4]. Southeast Asia, in particular, is considered a hot-spot for the generation of viruses that have zoonotic potential [5, 6]. This environment calls for increased surveillance for emerging diseases to help ensure that biosecurity measures are in place to protect humans, animals, and the environment. Hence, through this *One Health* pilot study set in Sarawak, Malaysia, we sought to conduct surveillance for respiratory and diarrheal pathogens that may potentially cross the species barrier between pigs and humans. More information on Sarawak can be found in the supplemental introduction (S1 Text). The primary aim of this study was to determine if viruses could be detected in bioaerosols collected at the animal-interface and to ascertain if these viruses were present in the nasal passages of humans working in these environments. Respiratory and swine pathogens with known or suspected transmission between pigs and humans were selected. More information on selection criteria can be found in the supplemental introduction (S1 Text). While finding molecular evidence of such animal viruses at the human-animal interface or in animal workers’ nasal passageways does not demonstrate active human infection, it does add to our understanding of the possible exposure risk. A secondary aim was to assess the understanding of zoonotic pathogens and their transmission to humans among animal workers, with the overall objective of aiding agriculture industries in controlling these pathogens in their livestock as well as informing cross-species infection prevention mechanisms.

## Methods

### Ethics

Laboratory assays were conducted under biosafety level 2 conditions, and field sampling methods were in compliance with protocols approved by the Institutional Animal Care and Use Committee of Duke University, as well as the Animal Care and Use Committee at the Ministry of Health Malaysia. Human survey and nasal wash collection protocols were approved by the Institutional Review Board at Duke University and the Medical Research and Ethics Committee at the Ministry of Health Malaysia. All enrolled human participants signed a written consent form, and animal farm managers gave verbal permission to collect samples from the pigs on their site.

### Site enrollment

Farms, abattoirs, and animal markets were identified and enrolled in Sibu and Kapit Divisions of Sarawak, Malaysia. All farms and abattoirs were visited once; each site manager of the farms was asked to complete a survey regarding site characteristics and biosecurity measures (supplemental information S1 Survey). Animal markets were visited on multiple occasions during June and July of 2017 to conduct participant enrollment with concurrent, localized bioaerosol sampling. Additional sampling was conducted to collect generalized bioaerosol samples from the market.

### Human, animal, and environmental sample collection

All employees present at their respective sites on the day of sampling were asked to participate. Employees were enrolled through face-to-face interaction, and were invited to participate if they were over the age of 18 and not pregnant. Participants were asked to complete a survey about their perceptions of cross-species infection risks and personal protective equipment (PPE) usage (supplemental information S2 Survey), as well as to permit collection of a nasal wash sample.

At each farm, up to five pig oral secretion or water trough samples and five pig fecal samples were collected. Fresh fecal specimens from the environment were swabbed and stored with a FecalSwab Collection, Transport and Preservation System (Copan Diagnostics, Inc., Murrieta, CA). Oral secretions were collected by allowing the pigs to chew on a cotton rope suspended from their housing structure as described previously [7–9]. Samples of standing water from the pigs’ feeders were collected in lieu of oral secretions for three farms with a local breed of domestic pig (babi kampung) that were averse to chewing the rope. Number, age, and gender of the pigs in the sampled enclosures were recorded.

Bioaerosol sampling was conducted using the National Institute for Occupational Safety and Health (NIOSH) BC 251 two-stage bioaerosol sampler [10] attached to an SKC personal air sampling pump and calibrated to 3.5 L/min of air flow. In farms and abattoirs, samplers were fixed to a tripod roughly one meter above the ground near an active pig pen and run for a minimum of 30 minutes. At the animal markets, mobile bioaerosol sampling localized to animal vendors where nasal washes were collected was conducted by a researcher equipped with a backpack containing the SKC pump with the NIOSH two-stage sampler fixed to one front strap for a minimum of one hour [11]. Mobile bioaerosol sampling was also conducted at the animal markets in the absence of other sampling types; this sampling was generalized, encircling the entire market to mimic a consumer walking up and down aisles of animal and vegetable products. Samples were processed within 24-hours of collection. Temperature and humidity were recorded from weather.com at the time of sampling.

More details regarding specimen collection are in the supplemental methods (S2 Text).

#### DNA/RNA extraction and RT-PCR

All samples were temporarily stored on ice after collection, then stored at -80°C until DNA and RNA extraction was performed. For each sample, 200 μL was extracted using the QIAmp Cador Pathogen Mini Kit (cat. 54106 QIAGEN) per the manufacturer instructions. Previously reported real-time polymerase chain reaction (rPCR) and real-time reverse transcription-polymerase chain reaction (rRT-PCR) probe-based assays for human adenovirus (ADV) [12], human coronavirus (CoV; multiplex assay) [13], encephalomyocarditis virus (EMCV) [14], panspecies enterovirus (EV) [15], influenza A, B, C, and D viruses (IAV, IBV, ICV, IDV) [16–18], porcine circovirus 2 (PCV2) [19], and porcine rotavirus A and C (RVA and RVC; multiplex assay) [20] were validated and performed with DNA or cDNA positive controls and nuclease free water negative controls using a BioRad CFx96 C1000 Touch Thermocycler Real Time detection system (primers and probe sequences are shown in supplemental S1 Table). The SsoAdvanced Universal Probes Supermix Real-Time PCR Kit (cat. 172–5281 BIORAD) was used for DNA viruses (ADV and PCV2), and SuperScript III Platinium One-Step qRT-PCR Kit (cat. 11732088 ThermoFisher) was used for RNA viruses (CoV, EMCV, EV, IAV-IDV, RVA, and RVC). A standard curve of known positive controls to validate the assay in laboratory was conducted prior to use in the field. A 38–40 C_T_ value range as suspect positive was used as a conservative cut-off to acknowledge that positivity at 38+ could be the result of cross-reactivity or non-specific amplification and thus have potential to be a false positive.

### Statistical analysis

Odds ratios and 95% confidence intervals were calculated for potential predictors of positivity of virus. Fisher’s exact test was used to measure statistical association of predictors for positivity of the viruses of interest amongst each of the sample types and were reported for variables determined to have a positive association at p<0.10. Statistical analyses were performed using STATA 15.0 (StataCorp, College Station, TX).

## Results

### Summary of enrollment sites

A total of 11 farms, 2 abattoirs, and 3 animal markets were enrolled in Sibu and Kapit Divisions of Sarawak, Malaysia in June and July of 2017. Eleven of the 12 registered commercial farms in Sibu were enrolled (site IDs F1 to F11), and one farm refused; the enrolled farms ranged from 0.12 to 2.43 hectares in size and housed a range of 100 to 7000 (median 350, IQR: 180, 850) pigs. All of the farms were open-air, and many housed other animals (including but not limited to cats, chickens, dogs, ducks, geese, ostriches, and turkeys) on the site. Biosecurity measures frequently reported by site managers included separate feed-in and waste-out paths (10 of 11 farms) and disinfecting of crates or pens between animals (8 of 10 farms, 1 missing). A Sibu abattoir (A1) slaughtered roughly 200 pigs nightly, bringing together pigs from many farms across Sarawak. An abattoir in Kapit (A2) was a more modest operation, slaughtering no more than five pigs per evening. Markets M1 and M2 were located in Sibu, and M3 was located in Kapit. The animal markets were all open-air and vendors sold both butchered meat (mainly pork and poultry), as well as live animals, including but not limited to chickens, ducks, rabbits, and puppies.

### Samples collected and viral prevalence

**Fig 1:**
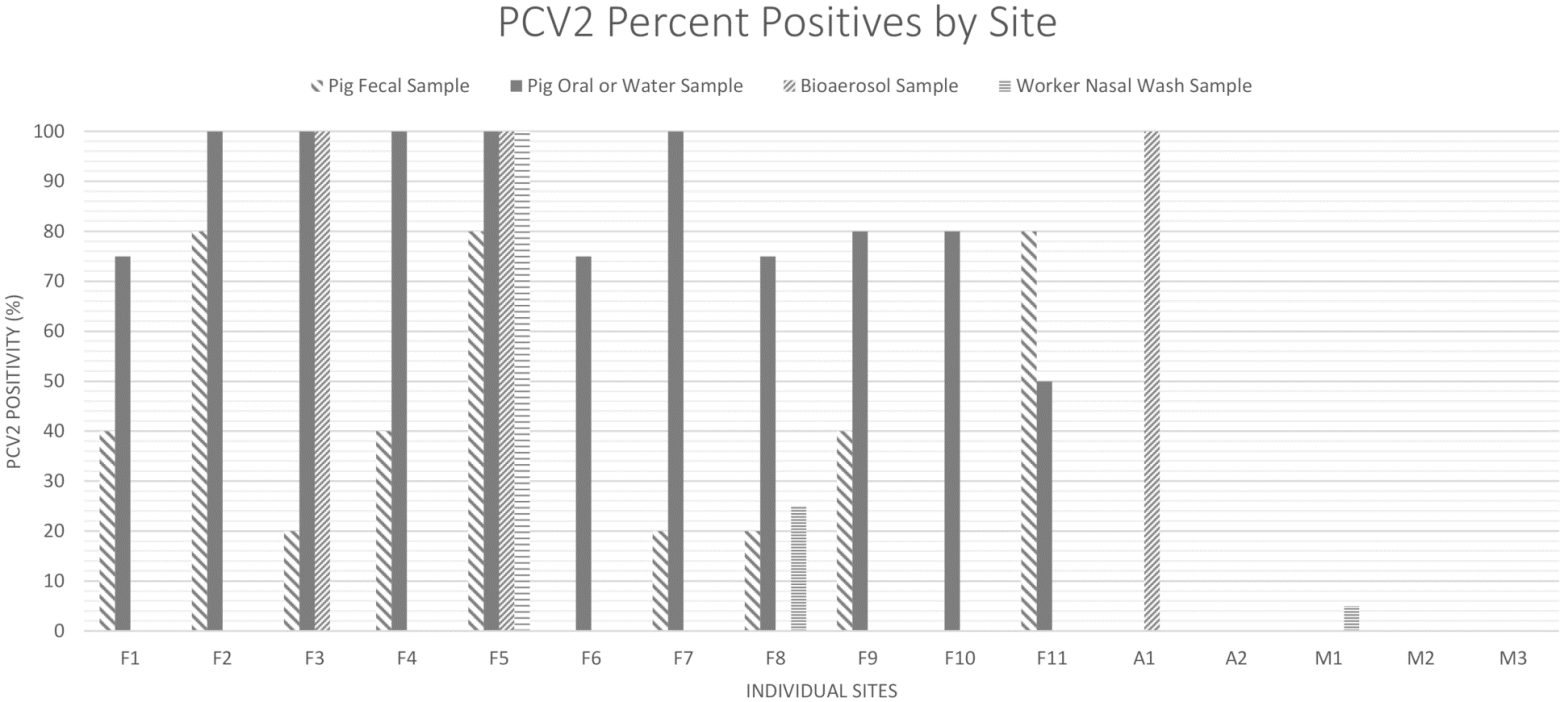
Molecular positivity for PCV2 detected via rPCR in samples collected from 11 farms, 2 abattoirs, and 3 live animal markets in Sarawak, Malaysia in June and July of 2017. Sites beginning with ‘F’ denotes farm, ‘A’ denotes abattoir, and ‘M’ denotes market; porcine circovirus 2 (PCV2).

At all sites, 78 out of 123 (63.4%) workers invited to participate agreed, and their nasal wash samples were collected. Further, 55 pig fecal, 49 pig oral or water, and 45 bioaerosol samples were collected across these sites (S2 Table). Of these, 21 (38.2%; average C_T_ 35.09) pig fecal, 43 (87.8%; average C_T_ 32.82) pig oral or water, 3 (14.2%; C_T_ 32.05, 34.20, and 36.19) bioaerosol, and 4 (5.1%; C_T_ 30.60, 36.00, 36.32, and 37.30) worker nasal wash samples were positive for PCV2 by rPCR (Table 1). PCV2 was detected in at least one sample at all 11 farms, as well as in a bioaerosol sample at one abattoir and one worker nasal wash at a market (Fig 1). Porcine RVC was detected in one pig fecal sample (1.8%; C_T_ 28.31). EMCV and porcine RVA were not detected in any of the sample types tested (Table 1). ADV was detected in three (3.8%; C_T_ 30.45, 32.20, and 35.03), CoV was detected in two (2.6%; C_T_ 25.50 and 37.84), and IBV (C_T_ 27.66) and IDV (C_T_ 16.18) were each detected in one (1.3%) worker nasal wash sample. IAV was detected in two (4.1%; C_T_ 35.19 and 38.48) pig oral secretions, and EV was detected in two (15.4%; C_T_ 36.60 and 36.71) bioaerosol samples. ICV was not detected in any of the sample types tested (Table 2). Of the 16 sites, 13 had at least one virus detected in any sample type, and 8 had positive results for more than one virus (S3 Table).

**Table 1:** Molecular positivity for swine viruses detected via rPCR or rRT-PCR in samples collected from animal environments in Sarawak, Malaysia in June and July of 2017.

Sample types (Total N)	% PCV2	% RVC	% RVA	% EMCV
Worker nasal wash (n = 78)	5.1	0	NT	0
Pig oral secretion or standing water sample(n = 49)	87.8	0	0	0
Pig fecal sample (n = 55)	38.2	1.8	0	0
Farm/abattoir bioaerosol sampling (n = 13)	23.1	0	0	0
Localized market bioaerosol sampling (n = 8)	0	NT	NT	NT

Porcine circovirus 2 (PCV2); porcine rotavirus C (RVC); porcine rotavirus A (RVA); encephalomyocarditis virus (EMCV); not tested (NT)

**Table 2:** Molecular positivity for respiratory viruses detected via rPCR or rRT-PCR in samples collected from animal environments in Sarawak, Malaysia in June and July of 2017.

Sample types (Total N)	% ADV	% CoV	% EV	% IAV	% IBV	% ICV	% IDV
Worker nasal wash (n = 78)	3.8)	2.6	0	0	1.3	0	1.3
Pig oral secretion or standing water sample(n = 49)	NT	NT	NT	4.1	0	0	0
Farm/abattoir bioaerosol sampling (n = 13)	0	0	15.4	0	0	0	0
Localized market bioaerosol sampling (n = 8)	0	0	0	0	0	0	0
Generalized market bioaerosol sampling (n = 24)	0	0	0	0	0	0	0

Adenovirus (ADV); coronavirus (CoV); enterovirus (EV); influenza A virus (IAV); influenza B virus (IBV); influenza C virus (ICV); influenza D virus (IDV); not tested (NT)

### Risk factor analysis

#### Pig viral shedding

While virus-positive pig oral secretions were not associated with the potential risk factors of pig breed and production type, there were associations with PCV2 detection in feces. The local pig breed, babi kampung, exhibited a higher prevalence of PCV2 results compared to standard domestic breeds (OR = 10.55, 95% C.I. 1.65, 110.87). Additionally, there was a nonstatistically significant trend towards an association of PCV2 in the feces for sows or gilts (breeding stock) as compared to other pig types (OR = 6.25, 95% C.I. 0.37, 353.48) (Table 3).

**Table 3:** Unadjusted odds ratios for risk factors associated with PCV2 molecular positivity of 55 pig fecal samples and 49 pig oral secretions or water samples collected from 11 pig farms in Sibu, Sarawak, Malaysia in July 2017.

	PCV2 in pig fecal samples			PCV2 in pig oral secretion or water samples		
Predictor	Total N	% Positive	Unadjusted OR (95% CI)	Total N	% Positive	Unadjusted OR (95% CI)
Pig breed	55	38.2		49	87.8	
Standard	40	27.5	Ref	37	89.2	2.06 (0.03, 29.37)
Babi kampung	10	80.0	10.55 (1.65, 110.87)	7	85.7	1.50 (0.02, 137.10)
Mix of breeds	5	40.0	1.76 (0.13, 17.39)	5	80.0	Ref
Production type	55	38.2		49	87.8	
Mix of >1 type	9	44.4	Ref	7	85.7	2.40 (0.09, 164.79)
Adult boar	3	0	0.00 (0.00, 2.28)	1	100.0	----
Adult sow or gilt	6	83.3	6.25 (0.37, 353.48)	10	100.0	----
Pregnant sow	15	20.0	0.31 (0.03, 2.75)	7	71.4	Ref
Farrowed sow	4	75.0	3.75 (0.18, 235.49)	1	100.0	----
Nursery	13	46.2	1.07 (0.14, 8.24)	15	86.7	2.60 (0.14, 43.45)
Finisher	5	0	0.00 (0.00, 1.31)	8	87.5	2.80 (0.11, 188.36)

Porcine circovirus 2 (PCV2)

#### Bioaerosol detection

Two of the three PCV2-positive bioaerosol samples were detected at farms, and one was detected at an abattoir. The two EV positive bioaerosol samples were detected at a farm and abattoir where PCV2 was also detected in bioaerosol samples. None of the bioaerosol samples collected in the animal markets were positive for the targeted viruses. All of the positive bioaerosol samples were found in the >4μm particle size fraction; there were no virus positives in found in the 1–4μm or <1μm particle size samples. When comparing these positive bioaerosol samples to others collected in close proximity to live animals (farm, abattoir, and localized market bioaerosol samples), neither temperature nor humidity were predictors of detection of PCV2 and EV (S4 Table). PCV2-positivity in these locations was not found to be a strong predictor of positivity in pig oral secretions or fecal samples; it also was not a statistically significant predictor of positivity in worker nasal washes, though the unadjusted OR was 2.5 (95% C.I. 0.03, 62.0) (S5 Table).

#### Worker nasal carriage of virus

PCV2 was the most commonly detected virus among human nasal washes, accounting for 4 of the 11 pathogen positive wash samples. Sixty-one of the 78 participants reported having close contact with pigs within one meter in the last 30 days; nine of the samples with any viral positivity, including all four of the participants that tested positive for PCV2, were among this category (Table 4). Interestingly, there was a greater prevalence of PCV2-positive results among study participants who had a household member who had contact with pigs within one meter in the last 30 days; although, this was not statistically significant (OR 9.43, 95% C.I. 0.57, 141.34) (Table 4).

**Table 4:** Unadjusted odds ratios (OR) for risk factors associated with molecular detection of virus in 78 worker nasal wash samples collected from farms, abattoirs, and live animal markets in Sarawak, Malaysia in June and July of 2017. Viral positivity is defined as a positive rPCRor rRT-PCR result for at least one of the following viruses: adenovirus, coronavirus, enterovirus, encephalomyocarditis virus, influenza A-D, porcine circovirus 2, or porcine rotavirus C.

		Viral positivity in worker nasal wash samples		PCV2 in worker nasal wash samples	
Risk factors	Total N polled	% Positive	Unadjusted OR (95% CI)	% Positive	Unadjusted OR (95% CI)
Contact with pigs (1 meter) in the last 30 days	78	14.1		5.1	
Yes	61	14.8	1.30 (0.23, 13.6)	6.6	Ref
No	17	11.8	Ref	0.0	0.00 (0.00, 3.45)
Household member contact with pigs (1 meter) in the last 30 days	77	14.3		5.2	
Yes	9	22.2	1.87 (0.16, 12.12)	22.2	9.43 (0.57, 141.34)
No	68	13.2	Ref	2.9	Ref
Type of pigs worked with in the last 30 days (>1 answer possible)	48	14.6		6.3	
Breeding	10	40.0	7.56 (0.95, 61.98)	10.0	1.94 (0.03, 40.75)
Farrowing	8	37.5	5.25 (0.57, 41.15)	0.0	0.00 (0.00, 6.61)
Nursery pigs	15	26.7	3.52 (0.49, 27.21)	13.3	4.77 (0.22, 290.18)
Wean-to-Finish	9	44.4	9.33 (1.12, 78.97)	11.1	2.25 (0.03, 47.40)
Finishers	8	37.5	5.25 (0.57, 41.15)	12.5	2.64 (0.04, 56.10)
Slaughter	18	5.6	0.24 (0.00, 2.28)	0	0.00 (0.00, 2.09)
Transport	17	17.6	1.39 (0.18, 9.50)	5.9	0.88 (0.01, 18.12)
Market stall type	30	13.3		3.3	
Butchered pork market stall	13	15.4	2.18 (0.10, 139.26)	7.7	----
Live chicken market stall	4	25.0	4.00 (0.04, 339.49)	0	
Butchered chicken market stall	13	7.7	Ref	0	
Total number of pigs on site (reported by site manager)	48	6.3		6.3	
1000+	7	42.9	10.88 (0.86, 153.20)	42.9	----
200–999	10	20.0	3.62 (0.22, 55.31)	0	
100–199	31	6.4	Ref	0	
Frequency of spotting rodents on farm or abattoir	46	13.0		4.4	
Daily	7	28.6	2.40 (0.09, 164.79)	28.6	----
Weekly	7	14.3	Ref	0	
Rarely	16	18.8	1.38 (0.09, 84.63)	0	
Never	16	0	----	0	

Porcine circovirus 2 (PCV2)

Looking more specifically at occupational exposures, there were significantly more virus positive results among those who reported working with wean-to-finish production (OR 9.33, 95% C.I. 1.12, 78.97) (Table 4). Though pig fecal sampling revealed greater PCV2 positivity among breeder females, there was no statistically significant difference in the number of PCV2-positive results among worker nasal washes with respect to working with different production types. There was no statistically significant difference in viral positivity among different market stall types (Table 4).

Finally, farm and abattoir characteristics were also examined as risk factors for viral positivity. While sites with a greater number of pigs presented a greater number of worker nasal wash PCV2-positive samples (all three farm worker nasal wash PCV2 positive samples were from farms with greater than 1000 head), this was not true for the full panel of viruses (Table 4). Additionally, a greater number of PCV2 positive results were observed on sites where workers reported seeing rodents on the site “daily”, as opposed to “weekly”, “rarely”, or “never” (Table 4). However, this trend was not observed for general viral positivity (Table 4).

### Worker perception of cross-species infection risk

When polled about how likely they think it is for people in Malaysia to become infected with “germs” from pigs and poultry, 46 of 76 (61%) participants who responded selected “likely” or “very likely”. Further, when polled about how likely they think it is for people in Sarawak to become infected with “germs” from pigs and poultry 48 of 76 (63%) participants who responded selected “likely” or “very likely”.

Of the 12 PPE suggestions, showering out before exiting the workplace and use of dedicated boots were the most cited methods of preventing cross-species infections and had the highest reported use (Table 5). Analysis comparing perception to use revealed four areas where the worker’s perception was not always associated with their use of PPE in the last 30 days. These practices included wearing protective glasses, receiving the flu vaccination, showering out of work, and wearing disposable booties. However, there was no obvious association of any of the 12 PPE use questions with molecular evidence of nasal carriage of virus.

**Table 5:** Perceptions of efficacy of personal protective equipment (PPE) at preventing cross-species infection, reported use, and viral positivity among PPE users in 77 worker nasal wash samples collected from farms, abattoirs, and live animal markets in Sarawak, Malaysia in June and July of 2017. Viral positivity is defined as a positive rPCR or rRT-PCR result for at least one of the following viruses: adenovirus, coronavirus, enterovirus, encephalomyocarditis virus, influenza A-D, porcine circovirus 2, or porcine rotavirus C.

Personal protective equipment	% Perceived as effective	% Used in the last 30 days	% Viral positivity among Users
Disposable gloves	29.8	13.0	20.0
Cloth gloves	19.5	19.5	0
Dust mask	44.2	6.5	20.0
Filtered mask	41.6	0	----
Glasses	15.6	5.2	50.0
Apron	32.5	15.6	8.3
Flu vaccination	55.8	5.2	25.0
Shower in	37.7	22.1	11.8
Shower out	92.2	96.1	14.9
Dedicated boots	72.7	53.2	14.6
Disposable boots	27.3	2.6	0

## Discussion

This report presents a first look at the prevalence of common swine respiratory and diarrheal viruses associated with swine environments in the Divisions of Sibu and Kapit, Sarawak, Malaysia. Some of these swine pathogens have potential to cross the species barrier from pigs to humans. Viruses detected by molecular means included ADV, CoV, EV, IAV, IBV, IDV, PCV2, and RVC among farms, abattoirs, and animal markets in Sarawak. Half of the sampled sites had more than one type of virus detected in the biological samples collected.

### Risk factors for viral positivity

#### Viral shedding in pigs

Previous literature suggests that the PCV2 virus is shed equally by oral and fecal routes in pigs [21, 22]. Our study, however, found a nearly 50% difference between fecal and oral samples, with oral secretions having the highest number of positive samples (though, it should be noted that the positive oral and fecal samples were not necessarily paired to the same animal). Interestingly, the local breed of pig babi kampung (concerning which there is little to no literature) had greater PCV2 viral shedding in feces compared to standard domestic breeds. We are uncertain why this was observed.

#### Aerosolization

PCV2 was detected in three (23.1%) farm/abattoir bioaerosol samples, and EV was detected in two (15.4%). However, positivity in bioaerosol samples was not well-correlated with positivity among other sample types. These results suggest that other types of environmental samples do not provide a reliable indication of possible bioaerosol exposures for workers in these settings. This could be due to differences in the aerosolization sources and rates, air velocities during sampling, environmental conditions, and other factors [23].

Bioaerosol sample concentrations are commonly found to have large variations both spatially and temporally, especially when there are point sources for the aerosols in the environment being studied [24–26]. While this study showed no notable difference in bioaerosol detection with respect to temperature and humidity, other studies have reported lower bioaerosol detection rates in hot and humid climates [9, 27]. At higher humidities, particles can become more hydrated which makes them larger and heavier and causes the particles to settle to the ground more quickly. Airborne particles larger than 4 μm are collected in the first stage of the NIOSH sampler, while 1–4 μm particles collect in the second stage and particles <1 μm collect on the filter [10]. It is noteworthy, then, that all of the positive samples were found only in the largest particle size collection tube, and the mean temperature and humidity recorded on sample dates were 29°C and 73%. Ventilation may make a difference in viral collection with these samplers. All of the bioaerosol samples were collected in open air environments without ventilation; therefore, lower airflow could also result in less aerosolization of virus. Additionally, it is possible that inhibitors (such as humic acids or phenolic compounds) were collected which may have introduced false negatives and skewed real-time PCR results [28].

#### Worker nasal carriage

Previous studies have reported mixed results regarding the zoonotic risk of PCV2 for those with occupational exposure to pigs or receiving a xenotransplantation [29–33]. While worker nasal carriage of virus could indicate aerosol transmission of virus, it is important to note that the presence of virus in a nasal wash could also be caused by handling contaminated items or fomites and self-inoculation of the nares. The sample size and number of virus-positive samples of worker nasal washes was small in our study, which limits our ability to perform a robust risk factor analysis. However, some trends do align with previous literature on cross-species infection risks and general farm biosecurity threats. For example, participants who reported a household member who had contact with pigs within one meter in the last 30 days had higher PCV2 positivity. This supports a common biosecurity practice on pig farms, asking that workers or visitors of the farm do not visit other farms or even interact with other pig farmers within a certain time period of returning to their own farms [34].

In addition to individual behaviors typically associated with higher viral carriage in farm workers, characteristics of the facility itself contribute to a higher risk of disease outbreaks or cross-species infection. In this study, PCV2 positivity was associated with larger herd sizes (1000 head or greater). Previous studies have also demonstrated that higher density herds may harbor more zoonotic viruses and generally be more prone to infections [35]. Sites where employees reported seeing rodents “daily”, as opposed to “weekly”, “rarely”, or “never”, were also more likely to have PCV2-positive results in humans. This is notable, as recent studies have shown molecular detection of PCV2 in rats and other rodents, suggesting cross-species transmission [36, 37]. While a positive association with rodents was observed for PCV2, this finding was not consistent when aggregating the nasal wash viral positive results.

### Perceptions and use of PPE

In addition to molecular detection of viruses, this study also presented a look at perceptions of biosecurity practices. Considering the 12 PPE questions, there were clear differences between perception and use for safety glasses, flu vaccinations, showering out of work, and disposable boots. While wearing safety glasses, flu vaccinations, and disposable boots were seen as efficacious but not used, showering out was often done by those who did not see it as an effective means of preventing cross-species infection. These differences could be due to training both for employers and workers, as the majority of farm biosecurity and disease prevention literature does not discuss worker protection against disease risks [34, 38, 39]. While not all 12 PPE items studied have proven effect in preventing cross-species infection or improving farm biosecurity, some listed interventions, such as rubber gloves, have shown marked differences in preventing the spread of zoonotic influenza virus [40].

### Study strengths and limitations

This study provides new prevalence data for some of the most economically important swine pathogens among 11 out of the 12 registered commercial farms in this seldom-studied region. This study also provides data for improving biosecurity practices in this region. However, this study had limitations. Cross-sectional studies such as this cannot prove causative relationships or examine seasonality of viruses. It is also limited via the lack of environmental controls and the lack of quantification of viruses. Additionally, the study’s sample size did not provide robust data for a confident risk factor analysis, especially among the human workers.

### Looking forward

More research is needed to understand differences in viral shedding among babi kampung. The bioaerosol samplers used in this study represent an underexplored technology that may provide effective, non-invasive surveillance for viruses in agricultural and many other settings. Serological studies will need to be done to assess the true risk of human infection with swine pathogens. The present study supports the feasibility of a seroepidemiological study of individuals with exposure to pigs in this region for PCV2 infection, as the virus appears to be ubiquitous in farms in Sibu. Future, more targeted studies in this region should include sequencing and phylogenetic analyses of viruses. Efforts to increase the use of PPE in agricultural settings in this region are needed, as well as more studies that aim to understand which PPE are most effective at preventing cross-species transmission of disease among those with occupational exposure to animals.

## Supporting information

S1 Text Supplemental introduction. (DOCX)

S2 Text Supplemental methods. (DOCX)

S1 Survey Sarawak animal worker study swine environment enrollment form. (DOCX)

S2 Survey Sarawak animal worker study employee specific behavior survey. (DOCX)

S1 Table Primer and probe sequences for rPCR and rRT-PCR. (DOCX)

S2 Table Number of biological and aerosol samples collected from 11 pig farms, 2 abattoirs, and 3 live animal markets in Sarawak, Malaysia in June and July of 2017. (DOCX)

S3 Table Molecular positivity for virus via rPCR or rRT-PCR in human, pig, or environmental samples collected from 11 farms, 2 abattoirs, and 3 live animal markets in Sarawak, Malaysia in June and July of 2017. (DOCX)

S4 Table Unadjusted odds ratios (OR) for risk factors associated with PCV2 and/or EV molecular positivity of 21 bioaerosol samples collected from 11 pig farms, 2 abattoirs, and 3 markets in Sarawak, Malaysia in July 2017. (DOCX)

S5 Table Unadjusted odds ratios (OR) for predictors associated with PCV2 positivity of 21 bioaerosol samples collected from 11 pig farms, 2 abattoirs, and 3 markets in Sarawak, Malaysia in July 2017. (DOCX)
