# Supplementary material for: Surveillance for respiratory and diarrheal pathogens at the human-pig interface in Sarawak, Malaysia
Source: PLoS One. 2018 Jul 27;13(7):e0201295. doi: 10.1371/journal.pone.0201295 (PMC6063427; doi:10.1371/journal.pone.0201295)
Supplement: S1 Text — (DOCX) [file pone.0201295.s001.docx]

**Surveillance for Respiratory and Diarrheal Pathogens at the Human-Pig Interface in Sarawak, Malaysia**

**S1 Text. SUPPLEMENTAL INTRODUCTION**

Sarawak is a state in Malaysia located on Borneo Island. It is a mixing pot of various ethnicities and cultures, and renowned for its biodiversity and unique flora and fauna. The two adjacent divisions of Sibu and Kapit located in the central zone of Sarawak, were selected for this pilot study. Sibu is located inland at the confluence of Rajang and Igan Rivers and covers an area of 8,278.3 Km² with a total population of 341,100 (1). Kapit, a larger division with mainly reserve forest located upriver from Sibu along the Rajang river, covers an area of 38,934 Km² with a population of 130,800 (1). A dense human population in Sibu and the abundance of natural resources in Kapit foster novel virus emergence and thus, the possibility of many zoonotic diseases.

A respiratory virus panel including influenza A, B, C and D (IAV, IBV, ICV & IDV), human adenovirus (AdV), human coronavirus (CoV), and panspecies enterovirus (EV) was selected for this study. IAV and IBV are two of the most common zoonotic respiratory viruses, as mentioned, with IAV having previously caused a major pandemic in 2009 (2). ICV and IDV are thought to be enzootic swine pathogens; according to the CDC, ICV has been known to cause mild infection in humans and IDV is not known to infect humans (3-4). Select samples were screened for AdV, CoV, and EV, as there is evidence of infection of multiple host species and, in some cases, cross-species transmission to humans (5-7).

Two swine disease experts, Dr. Alejandro Ramirez and Dr. Jim Lowe, suggested a panel of viruses that are important to the swine industry and may pose a threat to human health. Among those selected were porcine circovirus 2 (PCV2), porcine rotavirus C (porcine RVC), porcine rotavirus A (porcine RVA), and encephalomyocarditis virus (EMCV). While their roles in causing morbidity in pigs are well-documented, as similar viruses often infect humans, it is biologically plausible that animal-reservoired viruses may also infect humans, yet their zoonotic nature has not been comprehensively assessed. PCV2 has previously been studied for zoonosis risk via *in vitro* and *in vivo* experiments among patients who had undergone porcine islet cell transplantation and individuals with occupational exposure to pigs; however, these studies have presented mixed results.(8-12) Rotaviruses are known to infect many animals including pigs, cattle, and humans; some studies suggest similarities between certain porcine and human rotavirus strains(13), and cases of detection of porcine rotavirus in humans have recently been

reported (14). EMCV is also known to infect a variety of mammalian hosts, including humans (15-17). Transmission to humans is only known to occur through ingestion of food contaminated from an infected carcass; no direct transmission through contact of an infected animal has been reported (18).

**REFERENCES**

1. Department of Statistics, Malaysia. Current population estimates, Malaysia, 2004-2016. **2017**.

2. Centers for Disease Control and Prevention. First global estimates of 2009 H1N1 pandemic mortality released by CDC-led collaboration. Available at: <https://www.cdc.gov/flu/spotlights/pandemic-global-estimates.htm>. Accessed 11 January 2018.

3. Centers for Disease Control and Prevention. *Types of influenza viruses*. Available at: <https://www.cdc.gov/flu/about/viruses/types.htm>. Accessed 11 January 2018.

4. Su S, Fu X, Li G, Kerlin F, Veit M. Novel Influenza D virus: Epidemiology, pathology, evolution and biological characteristics. Virulence. 2017 Nov 17;8(8):1580-91.

5. Su S, Wong G, Shi W, Liu J, Lai AC, Zhou J, et al. Epidemiology, genetic recombination, and pathogenesis of coronaviruses. Trends in microbiology. 2016 Jun 1;24(6):490-502.

6. Wevers D, Metzger S, Babweteera F, Bieberbach M, Boesch C, Cameron K, et al. Novel adenoviruses in wild primates: a high level of genetic diversity and evidence of zoonotic transmissions. Journal of Virology. 2011 Oct 15;85(20):10774-84.

7. Krauss H, Schieffer HG, Slenczka W. Zoonoses: infectious diseases transmissible from animals to humans. American Society for Microbiology; 2003.

8. Burbelo PD, Ragheb JA, Kapoor A, Zhang Y. The serological evidence in humans supports a negligible risk of zoonotic infection from porcine circovirus type 2. Biologicals **2013**; 41(6): 430-4.

9. Allan GM, McNeilly F, McNair I, Curran MD, Walker I, Ellis J, et al. Absence of evidence for porcine circovirus type 2 in cattle and humans, and lack of seroconversion or lesions in experimentally infected sheep. Archives of Virology **2000**; 145(4): 853-7.

10. Ellis JA, Wiseman BM, Allan G, Konoby C, Krakowka S, Meehan BM, et al. Analysis of seroconversion to porcine circovirus 2 among veterinarians from the United States and Canada. Journal of the American Veterinary Medical Association **2000**; 217(11): 1645-6.

11. Tischer I, Bode L, Apodaca J, Timm H, Peters D, Rasch R, et al. Presence of antibodies reacting with porcine circovirus in sera of humans, mice, and cattle. Archives of Virology **1995**; 140(8): 1427-39.

12. Li L, Kapoor A, Slikas B, Bamidele OS, Wang C, Shaukat S, et al. Multiple diverse circoviruses infect farm animals and are commonly found in human and chimpanzee feces. Journal of Virology **2010**; 84(4): 1674-82.

13. Wakuda M, Ide T, Sasaki J, Komoto S, Ishii J, Sanekata T, et al. Porcine rotavirus closely related to novel group of human rotaviruses. Emerging Infectious Diseases **2011**; 17(8): 1491.

14. Wu FT, Bányai K, Jiang B, Liu LT, Marton S, Huang YC, et al. Novel G9 rotavirus strains co-circulate in children and pigs, Taiwan. Scientific Reports **2017**; 7.

15. Murnane TG, Craighead JE, Mondragon H, Shelokov A. Fatal disease of swine due to encephalomyocarditis virus. Science **1960**; 131(3399): 498-9.

16. Wells SK, Gutter AE, Soike KF, Baskin GB. Encephalomyocarditis virus: epizootic in a zoological collection. Journal of Zoo and Wildlife Medicine **1989**: 291-6.

17. Oberste MS, Gotuzzo E, Blair P, Nix WA, Ksiazek TG, Comer JA, et al. Human febrile illness caused by encephalomyocarditis virus infection, Peru. Emerging Infectious Diseases **2009**; 15(4): 640.

18. Swine Health Information Center. Encephalomyocarditis virus. Available at: <http://www.swinehealth.org/wpcontent/uploads/2016/03/Encephalomyocarditis-virus-EMCV.pdf>. Accessed 11 January 2018.
