# Supplementary material for: Surveillance for respiratory and diarrheal pathogens at the human-pig interface in Sarawak, Malaysia
Source: PLoS One. 2018 Jul 27;13(7):e0201295. doi: 10.1371/journal.pone.0201295 (PMC6063427; doi:10.1371/journal.pone.0201295)
Supplement: S2 Text — (DOCX) [file pone.0201295.s002.docx]

**Surveillance for Respiratory and Diarrheal Pathogens at the Human-Pig Interface in Sarawak, Malaysia**

**S2 Text. SUPPLEMENTAL METHODS**

**Worker Nasal Wash**

For nasal wash sample collection, participants were asked to tilt their head to a 70-degree angle and briefly hold their breath while one nostril was irrigated with 3mL of sterile water using a 10mL plastic syringe. The participant then tilted their head back down and expressed the fluid into a sterile specimen collection cup. The return fluid was transferred into an Olympus 2.0ml screw-cap microtube. Collection cups containing low yield samples (<1mL) were rinsed with an additional 500μL of sterile water to achieve sufficient volume to pipette.

**Pig Oral Secretion**

Two 30cm pieces of 1cm diameter 100% cotton rope were tied to the housing structure of the pigs at a height within reach of the animals’ mouths. Pigs were allowed to chew on the rope until saturation was achieved before the rope was removed aseptically with a sterile plastic bag. A corner of the bag was clipped, and the rope was wrung manually through the bag into an Olympus 2.0ml screw-cap microtube. Low yield samples (<1mL) were supplemented up to 1mL with PBS.

**Bioaerosol**

Aerosols collected in the greater than 4µm and 1 to 4µm fractions were processed by adding 1mL of sterile PBS with 0.5% BSA, followed by thorough inversion and vortexing. For aerosols less than 1µm, the filter was removed from the cassette carefully and transferred with forceps into a 50mL Falcon tube with 2.0mL of PBS with 0.5% BSA. The Falcon tube was vortexed, and the filter was removed with forceps and discarded. The remaining liquid was transferred into an Olympus 2.0ml screw-cap microtube.
