# Supplementary material for: Surveillance for respiratory and diarrheal pathogens at the human-pig interface in Sarawak, Malaysia
Source: PLoS One. 2018 Jul 27;13(7):e0201295. doi: 10.1371/journal.pone.0201295 (PMC6063427; doi:10.1371/journal.pone.0201295)
Supplement: S1 Survey — (DOCX) [file pone.0201295.s003.docx]

**S1 SURVEY. Sarawak animal worker study Site ID:**

Swine environment enrollment form

Today’s date (day/month/year): ___/___/_____

**How many square kilometers is the site on?** ______square Km

**What is the average number of animals on a single day at the site?** ____

**How many individuals are employed at the site?** ______

**Is there another pig farm located within a 2 kilometer radius of the site?**


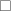
 Yes
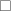
 No
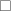
 Unknown

**Are there any of the following animals located within a 100m radius of the farm? (Check all that apply)**


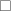
 Cattle
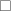
 Sheep
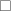
 Cats
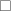
 Dogs
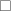
 None

**Does the facility have an indoor water source available?**


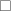
 Yes
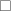
 No
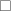
 Unknown

**Are the facilities kept well-ventilated and dry?**


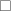
 Yes
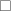
 No
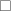
 Unknown

**Sarawak animal worker study Site ID:**

Swine environment enrollment form

Today’s date (day/month/year): ___/___/_____

**If your facility is a farm:**

**Does the site use any of the following biosecurity measures?**

**(Check all that apply)**


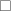
 “all in, all out” production


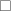
 clothing or equipment is cleaned/disinfected/discarded after use


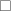
 working clothes cannot be worn off farm site


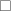
 structures are made of steel or plastic (as opposed to wood surfaces)


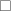
 transport cages are made of steel or plastic (as opposed to wood surfaces)


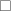
 crates/pens are cleaned/disinfected between animals


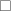
 separated feed in-paths and feces-out paths


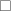
 treatment of water from ponds or dams


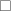
 farm windows closed


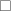
 wild birds actively kept away from animals


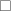
 vaccination of animals to swine influenza


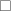
 segregated structure for sick animals


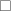
 euthanization of sick animals

**If your facility is a slaughter house:**

**Does the site use any of the following biosecurity measures?**

**(Check all that apply)**


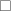
 clothing or equipment is cleaned/disinfected/discarded after use


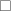
 pens are cleaned/disinfected after each farm


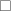
 utensils for meat processing are cleaned/disinfected between each farm


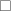
 working clothes cannot be worn off site


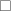
 site windows are kept closed


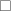
 structures are made of steel or plastic as opposed to wood/uncleanable surfaces
