# Supplementary material for: Surveillance for respiratory and diarrheal pathogens at the human-pig interface in Sarawak, Malaysia
Source: PLoS One. 2018 Jul 27;13(7):e0201295. doi: 10.1371/journal.pone.0201295 (PMC6063427; doi:10.1371/journal.pone.0201295)
Supplement: S2 Survey — (DOCX) [file pone.0201295.s004.docx]

**S2 SURVEY. Sarawak animal worker study Site ID:**

Employee specific behavior survey **Participant ID:**

Today’s date (day/month/year): ___/___/_____

**Perceptions of cross-species infection and PPE use**

|  | Pigs | Chickens | Ducks | Geese | Other Poultry | Other Wildlife |
| --- | --- | --- | --- | --- | --- | --- |
| In the last 30 days have you come in close contact (touching or within 1 meter) with these animals (Check all that apply): |  |  |  |  |  |  |
| In the last 12 months have you come in close contact (touching or within 1 meter) with these animals (Check all that apply): |  |  |  |  |  |  |
|  |  |  |  |  |  |  |
|  |  |  |  |  |  |  |
| In the last 30 days has anyone living in your household had close contact (touching or within 1 meter) with any of the following animals (Check all that apply): |  |  |  |  |  |  |

|  | Very Unlikely | Unlikely | Neutral | Likely | Very Likely |
| --- | --- | --- | --- | --- | --- |
| How likely do you think it is for people in Malaysia to become infected with germs from pigs or poultry? (Please choose 1) |  |  |  |  |  |
|  |  |  |  |  |  |
|  |  |  |  |  |  |
| How likely do you think it is for people in Sarawak to become infected with germs  from pigs or poultry? (Please choose 1) |  |  |  |  |  |
|  |  |  |  |  |  |
|  |  |  |  |  |  |

**Which of these equipment/measures do you consider protective against cross-species infections? (Check all that apply)**


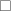
 disposable latex or vinyl gloves
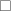
 influenza vaccination


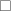
 cloth or leather gloves
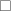
 shower in


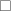
 dust/particle mask
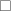
 shower out


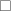
 filtered mask
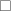
 dedicated boots


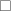
 glasses/eye protection
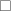
 disposable booties


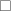
 apron
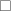
 dedicated clothing

**What types of equipment or measures have you used while working with animals in the last 30 days? (Check all that apply)**


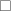
 disposable latex or vinyl gloves
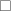
 influenza vaccination


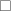
 cloth or leather gloves
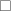
 shower in


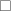
 dust/particle mask
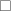
 shower out


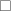
 filtered mask
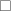
 dedicated boots


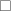
 glasses/eye protection
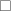
 disposable booties


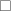
 apron
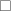
 dedicated clothing

**Sarawak animal worker study Site ID:**

Employee specific behavior survey **Participant ID:**

Today’s date (day/month/year): ___/___/_____

**Animal market only**

**What type of work have you performed with animals in the last 30 days?**

**(Check all that apply)**


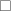
 General husbandry (i.e. feeding)
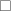
 Euthanizing diseased/injured animals


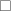
 Transporting animals
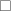
 Slaughtering and/or butchering animals


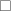
 Treating diseased animals
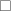
 Packaging raw meat products


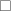
 Obtaining blood samples from animals

**Do you use halal practices for animal slaughter?**


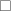
 Yes
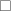
 No
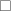
 Not Applicable

**Swine environments only**

**How many years have you been employed as a swine worker?** ______ years

**What type of work have you performed with pigs in the last 30 days?**

**(Check all that apply)**


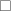
 General husbandry (i.e. feeding)
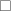
 Obtaining blood samples from pigs


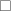
 Disinfecting crates/pens
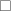
 Treating diseased animals


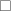
 Transporting pigs
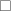
 Euthanizing diseased/injured animals


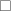
 Breeding stock
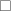
 Slaughtering and/or butchering pigs


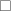
 Assisting with birth
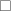
 Packaging raw pork products

**What production type of pigs do you most commonly work with?**

**(Check all that apply)**


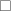
 Nursery
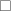
 Farrowing


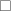
 Finishing
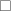
 Wean-to-Finish


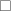
 Breeding
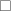
 Slaughter only

**How frequently are rodents (mice/rats) spotted on the site?**


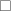
 Daily
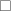
 Once per week
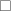
 Rarely
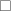
 Never
