# Supplementary material for: Surveillance for respiratory and diarrheal pathogens at the human-pig interface in Sarawak, Malaysia
Source: PLoS One. 2018 Jul 27;13(7):e0201295. doi: 10.1371/journal.pone.0201295 (PMC6063427; doi:10.1371/journal.pone.0201295)
Supplement: S1 Table — (DOCX) [file pone.0201295.s005.docx]

**S1 Table: Primer and probe sequences for rPCR and rRT-PCR**

|  | **Forward primer(s)** | **Reverse primer(s)** | **Probe(s)** | **Gene or Target** | **Reference** |
| --- | --- | --- | --- | --- | --- |
| **ADV** | 5’-CAG-GAC-GCY-TCG-GAG-TAC-CTG-A-3’ | 5’-CGG-TGG-TCA-CAT-CGT-GGG-T-3’  5’-GCT-GAA-GTA-CGT-VTC-GGT-GGC-3’  5’-GGT-GAA-GTA-GGT-GTC-CGT-GGC-3’ | 5’-FAM-TGG-TGC-AGT-TYG-CCC-G-MGB(NFQ)-3’ | Hexon | Bil-Lula *et al.*, 2012 |
| **CoV** | 5’-GTTCTGATAAGGCACCATATAGG-3’  5’-CATACTCTGACGGTCACAATAATA-3’  5’-TCCTACTAYTCAAGAAGCTATCC-3  5’-CATACTATCAACCCATTCAACAAG-3’ | 5’-TTTAGGAGGCAAATCAACACG-3’  5’-ACCTTAGCAACAGTCATATAAGC-3’  5’-AATGAACGATTATTGGGTCCAC-3’  5’-CACGGCAACTGTCATGTATT-3’ | 5’-TXR-CGCATACGCCAACGCTCTTGAACA-3’  5’-YAK-TGCCCAAGAATAGCCAGTACCTAGT-3’  5’-CY5-TYCGCCTGGTACGATTTTGCCTCA-3’  5’-FAM-ATGAACCTGAACACCTGAAGCCAATCTATG-3’ | NL63, OC43, HKU1, 229E | Loens *et al*., 2012 |
| **EMCV** | 5’-TCA TTA GCC ATT TCA ACC CA-3’ | 5’-GAG ATA CAA ACC CGC CCT AA--3’ | 5’-FAM-TCC CAT CAG GTT GTG CAG CGA-TAMRA-3’ | 3D | Yuan *et al*., 2014 |
| **EV** | 5’-GGCCCCTGAATGCGGCTAATCC-3’ | 5’-GCGATTGTCACCATWAGCAGYCA-3’ | 5’-FAM-CCGACTACTTTGGGWGTCCGTGT-IBFQ-3’ | 5’NTR | Oberste *et al.*, 2012 |
| **IAV** | 5’-GAC-CRA-TCC-TGT-CAC-CTC-TGA-C-3’ | 5’-AGG-GCA-TTY-TGG-ACA-AAK-CGT-CTA-3’ | 5’-FAM-TGC-AGT-CCT-CGC-TCA-CTG-GGC-ACG-BHQ 1-3’ | Matrix | Centers for Diseases Control and Prevention, 2017 |
| **IBV** | 5’-TCC-TCA-AYT-CAC-TCT-TCG-AGC-G-3’ | 5’-CGG-TGC-TCT-TGA-CCA-AAT-TGG-3’ | 5’-FAM-CCA-ATT-CGA-GCA-GCT-GAA-ACT-GCG-GTG-BHQ 1-3’ | Matrix | Centers for Disease Control and Prevention, 2017 |
| **ICV** | 5’-TGGGAGAGATGGTGTGGAGATA-3’ | 5’-TCTTTTTCCATCGAGTCAATTTCA-3’ | 5’-FAM-AAAGACCACAATTATGC-IBFQ-3’ | Matrix | Hause *et al*., 2013 |
| **IDV** | 5’-GCTGTTTGCAAGTTGATGGG-3’ | 5’-TGAAAGCAGGTAACTCCAAGG-3’ | 5’-FAM-TTCAGGCAAGCACCCGTAGGATT-IBFQ-3’ | C/OK | Pabbaraju *et al*., 2013 |
| **PCV2** | 5’-TGG CCC GCA GTA TTC TGA TT -3’ | 5’-CAG CTG GGA CAG CAG TTG AG-3’ | 6FAM-5’-CCA GCA ATC AGA CCC CGT TGG AAT G-3′-  TAMRA | 1561-1632bp | Pal *et al*., 2008 |
| **RVA** | 5’-GCT AGG GAY AAA ATT GTT GAA GGT A-3’ | 5’-ATT GGC AAA TTT CCT ATT CCT CC-3’ | 5’-FAM-ATG AAT GGA AAT GAY TTT CAA AC-MGB--3’  5’-FAM-ATG AAT GGA AAT AAT TTT CAA AC-MGB-3’ | VP6 | Marthaler *et al*., 2014 |
| **RVC** | 5’-ATG TAG CAT GAT TCA CGA ATG GG-3’ | 5’-ACA TTT CAT CCT CCT GGG GAT C-3’ | 5’-HEX-GCG TAG GGG CAA ATG CGC ATG A-TAMRA-3’ | VP6 | Marthaler *et al*., 2014 |

adenovirus (ADV); coronavirus (CoV); encephalomyocarditis virus (EMCV); enterovirus (EV); influenza A virus (IAV); influenza B virus (IBV); influenza C virus (ICV); influenza D virus (IDV); porcine circovirus 2 (PCV2); porcine rotavirus A (RVA); porcine rotavirus C (RVC).
