# Supplementary material for: Surveillance for respiratory and diarrheal pathogens at the human-pig interface in Sarawak, Malaysia
Source: PLoS One. 2018 Jul 27;13(7):e0201295. doi: 10.1371/journal.pone.0201295 (PMC6063427; doi:10.1371/journal.pone.0201295)
Supplement: S2 Table — (DOCX) [file pone.0201295.s006.docx]

| **Site IDs** | Pig fecal samples | | Pig oral secretion or water samples | Bioaerosol samples | Worker nasal washes |
| --- | --- | --- | --- | --- | --- |
| F1 | | 5 | 4 | 1 | 2 |
| F2 | | 5 | 5 | 1 | 1 |
| F3 | | 5 | 5 | 1 | 3 |
| F4 | | 5 | 5 | 1 | 2 |
| F5 | | 5 | 5 | 1 | 2 |
| F6 | | 5 | 4 | 1 | 2 |
| F7 | | 5 | 5 | 1 | 1 |
| F8 | | 5 | 4 | 1 | 4 |
| F9 | | 5 | 5 | 1 | 1 |
| F10 | | 5 | 5 | 1 | 1 |
| F11 | | 5 | 2 | 1 | 1 |
| A1 | | - | - | 1 | 25 |
| A2 | | - | - | 1 | 3 |
| M1 | | - | - | 27 | 20 |
| M2 | | - | - | 3 | 2 |
| M3 | | - | - | 2 | 8 |
| Total | | 55 | 49 | 45 | 78 |

**S2 Table: Number of biological and aerosol samples collected from 11 pig farms, 2 abattoirs, and 3 live animal markets in Sarawak, Malaysia in June and July of 2017.**

Sites beginning with ‘F’ denotes farm, ‘A’ denotes abattoir, and ‘M’ denotes market.
