# Supplementary material for: Surveillance for respiratory and diarrheal pathogens at the human-pig interface in Sarawak, Malaysia
Source: PLoS One. 2018 Jul 27;13(7):e0201295. doi: 10.1371/journal.pone.0201295 (PMC6063427; doi:10.1371/journal.pone.0201295)
Supplement: S3 Table — (DOCX) [file pone.0201295.s007.docx]

| **Site ID** | ADV | CoV | EMCV | EV | IAV | IBV | ICV | IDV | PCV2 | RVA | RVC |
| --- | --- | --- | --- | --- | --- | --- | --- | --- | --- | --- | --- |
| F1 | - | + | - | - | - | - | - | - | + | - | - |
| F2 | - | - | - | - | - | - | - | - | + | - | - |
| F3 | - | - | - | + | - | - | - | - | + | - | - |
| F4 | - | - | - | - | - | - | - | - | + | - | - |
| F5 | - | - | - | - | - | - | - | - | + | - | - |
| F6 | - | - | - | - | + | - | - | - | + | - | - |
| F7 | + | - | - | - | - | - | - | - | + | - | - |
| F8 | - | - | - | - | - | - | - | - | + | - | - |
| F9 | - | - | - | - | - | - | - | - | + | - | + |
| F10 | - | - | - | - | - | - | - | - | + | - | - |
| F11 | - | - | - | - | - | + | - | - | + | - | - |
| A1 | + | - | - | + | - | - | - | - | + | - | - |
| A2 | - | - | - | - | - | - | - | - | - | - | - |
| M1 | + | + | - | - | - | - | - | + | + | - | - |
| M2 | - | - | - | - | - | - | - | - | - | - | - |
| M3 | - | - | - | - | - | - | - | - | - | - | - |

**S3 Table: Molecular positivity for virus via rPCR or rRT-PCR in human, pig, or environmental samples collected from 11 farms, 2 abattoirs, and 3 live animal markets in Sarawak, Malaysia in June and July of 2017.**

Sites beginning with ‘F’ denotes farm, ‘A’ denotes abattoir, and ‘M’ denotes market; adenovirus (ADV); coronavirus (CoV); encephalomyocarditis virus (EMCV); enterovirus (EV); influenza A virus (IAV); influenza B virus (IBV); influenza C virus (ICV); influenza D virus (IDV); porcine circovirus 2 (PCV2); porcine rotavirus A (RVA); porcine rotavirus C (RVC).
