# Supplementary material for: Surveillance for respiratory and diarrheal pathogens at the human-pig interface in Sarawak, Malaysia
Source: PLoS One. 2018 Jul 27;13(7):e0201295. doi: 10.1371/journal.pone.0201295 (PMC6063427; doi:10.1371/journal.pone.0201295)
Supplement: S4 Table — (DOCX) [file pone.0201295.s008.docx]

**S4 Table: Unadjusted odds ratios (OR) for risk factors associated with PCV2 and/or EV molecular positivity of 21 bioaerosol samples collected from 11 pig farms, 2 abattoirs, and 3 markets in Sarawak, Malaysia in July 2017.** Note: both sites with EV positives were also positive for PCV2.

|  |  | **Viral positivity in bioaerosol samples** | |
| --- | --- | --- | --- |
| **Risk factors** | **Total N** | **No. (%)** | **Unadjusted OR**  **(95% CI)** |
| Outside temperature (^o^C) | 21 | 3 (14.3) |  |
| 24-27 | 8 | 2 (28.6) | 1.67 (0.06, 117.71) |
| 28-30 | 6 | 1 (14.3) | Ref |
| 30-34 | 7 | 0 (0.0) | ---- |
| Outside humidity (%) | 21 | 3 (14.3) |  |
| 52-69 | 7 | 0 (0.0) | ---- |
| 70-81 | 8 | 1 (12.5) | Ref |
| 81-99 | 6 | 2 (33.3) | 3.50 (0.13, 235.39) |

Porcine circovirus 2 (PCV2); enterovirus (EV)
