# Supplementary material for: Surveillance for respiratory and diarrheal pathogens at the human-pig interface in Sarawak, Malaysia
Source: PLoS One. 2018 Jul 27;13(7):e0201295. doi: 10.1371/journal.pone.0201295 (PMC6063427; doi:10.1371/journal.pone.0201295)
Supplement: S5 Table — (DOCX) [file pone.0201295.s009.docx]

**S5 Table: Unadjusted odds ratios (OR) for predictors associated with PCV2 positivity of 21 bioaerosol samples collected from 11 pig farms, 2 abattoirs, and 3 markets in Sarawak, Malaysia in July 2017.**

|  |  | **PCV2 positivity in bioaerosol samples** | |
| --- | --- | --- | --- |
| **Predictor** | **Total N** | **No. (%)** | **Unadjusted OR**  **(95% CI)** |
| Pig oral secretion/water sample PCV2 positivity | 11 | 2 (18.2) |  |
| Positive | 11 | 2 (18.2) | ---- |
| Negative | 0 | 0 (0.0) |  |
| Pig fecal sample PCV2 positivity | 11 | 2 (18.2) |  |
| Positive | 9 | 2 (22.2) | Ref |
| Negative | 2 | 0 (0.0) | 0.00 (0.00, 10.63) |
| Worker nasal wash sample PCV2 positivity | 21 | 3 (14.3) |  |
| Positive | 4 | 1 (25.0) | 2.5 (0.03, 62.0) |
| Negative | 17 | 2 (11.8) | Ref |

Porcine circovirus 2 (PCV2)
